# Influencing factors of urban safety perception based on the combination of multi-source data and machine learning: a case study of Nanchang City, China

**DOI:** 10.1038/s41598-025-34110-3

**Published:** 2025-12-30

**Authors:** Linduo Yuan, Yuze Dan, Yi Leng, Jiaxin Zhang

**Affiliations:** 1 College of Architecture and Urban Planning, Guizhou University, Guizhou, 550025 China; 2 School of Architecture and Urban Planning, Chongqing University, Chongqing, 400044 China; 3 Key Laboratory of New Technology for Construction of Cities in Mountain Area, Chongqing University, Chongqing, 400044 China; 4 Architecture and Design College, Nanchang University, No.999, Xuefu Avenue, Honggutan New District, Nanchang, 330031 China

**Keywords:** Urban safety perception, SHAP machine learning, Multi-source data, Psychological perception, Nan chang, Development studies, Environmental studies, Geography, Geography, Sociology

## Abstract

Research examines the influence of multisource urban data on residents’ perceptions of safety. Utilizing the SHAP machine learning model, the research conducts a comprehensive analysis of the nonlinear relationships and interactive effects between built environment factors and psychological perception factors on urban residents’ safety perceptions. Focusing on Nanchang City as a case study, the research integrates multidimensional data encompassing urban spatial environments, resident perceptions, and socioeconomic indicators. The findings highlight the critical role of perceived urban vitality and perceived wealth in shaping residents’ safety perceptions, addressing the insufficient consideration of individual psychological factors in previous research, this study innovatively incorporates psychological perception data, thereby extending traditional built environment theories. By employing nonlinear models to elucidate the influence mechanisms of different variables across spatial zones, it provides a scientific foundation for urban planning and safety governance. Additionally, selecting Nanchang, a representative medium-sized city characterized by historical and cultural heritage, as the sample addresses the previous research gap in such urban contexts. By establishing an evaluation framework for urban safety perception based on multi-source data, this study offers theoretical support and practical guidance for precision planning in medium-sized cities dominated by historical and cultural heritage. This contributes to advancing sustainable urban development and enhancing residents’ well-being.

## Introduction

Cities serve as vital spatial carriers for human existence, providing residents with essential material and psychological security^1^. As urbanization progresses, the residents’ perception of safety increasingly emerges as a critical indicator of livability and social cohesion^5^. In the context of stock urbanization, investigating urban safety perception holds substantial value for guiding urban renewal and the transformation into age-friendly cities. ^6^ The perception of urban safety pertains not only to the fundamental survival security of city residents but also influences their mental health and the sustainable development of the city.

Research on urban safety perception initially concentrated on crime prevention and environmental design. In the early 20th century, the international academic community began to explore the concept of a ‘sense of safety’ within urban spaces, proposing theories to enhance residents’ subjective experience of safety through urban planning, crime control, and environmental design. Among these, Jacobs^7^ introduced the ‘eyes on the street’ theory in *The Death and Life of Great American Cities*, emphasizing the importance of street vitality and diversity in enhancing the sense of safety. Furthermore, Wilson and Kelling^8^ in *Broken Windows* indicated that environmental cleanliness and order significantly influence residents’ sense of security.

As early research primarily relied on field survey data and theoretical analysis, it was challenging to systematically collect and analyze residents’ safety perception data within a multidimensional framework. In recent years, with the development of CGSS and multi-source urban data, research has increasingly shifted towards quantifying and analyzing multidimensional data on urban space and the built environment to reveal the interactive effects of various influencing factors on residents’ perceptions of urban safety. Y. Zhang et al. ^9^ through empirical analysis of 278 urban communities nationwide, established a multilevel linear model to examine the impact of China’s built environment on residential safety perceptions. They questioned the limitations of the “eyes on the street” ^7^protection mechanism, proposing that mixed-use, dense road networks, and compact built environments exert a significant negative influence on individual residential safety perceptions. ^9^ Bao and Yang^10^ utilized image semantic segmentation techniques to identify three key elements—traffic spaces, street environments, and spatial interfaces—as having a pronounced effect on perceived visual safety. Qiu et al.^11^ similarly analyzed Shanghai’s street imagery and property price data, discovering that green coverage and street design exert a significant positive influence on residents’ safety perceptions. Furthermore, research by Zeng et al. ^12^indicates that social factors and physical activities within the built environment exert multiple pathways of influence on residents’ sense of security.

With the advancement of big data and machine learning technologies, the utilization of multi-source data in urban safety perception research has become increasingly prevalent. International studies have adopted multi-source big data technologies earlier, employing tools such as street view imagery, social media text, and mobile phone data to conduct quantitative assessments of urban safety perceptions. Naik et al. ^13^ utilized machine learning and image recognition techniques to extract visual elements influencing perceptions from urban street scenes, thereby exploring the relationship between environmental characteristics and residents’ sense of safety. Domestically(In China), the rapid advancement of big data, deep learning, and spatial information technologies has gradually shifted research away from traditional questionnaire surveys towards empirical analysis using quantitative assessment methods that integrate multi-source data. For instance, Qin et al.^5^ employed multi-source big data to construct a spatial distribution model of urban safety perception in Nanjing’s central urban area, systematically evaluating the spatial patterns and influencing mechanisms of urban safety perception across three dimensions: individual perception, built environment, and behavioral activities. Furthermore, street-view image analysis and interpretable machine learning models have been applied to identify spatial characteristics influencing perceptions. Li X. et al.^14^ employed image semantic segmentation to extract visual elements from street views, constructing a road safety perception dataset through manual scoring combined with deep learning. Machine learning was then utilized to identify the visual factors affecting environmental safety perceptions, revealing the role of elements such as open sightlines, green space layout, and spatial connectivity in shaping residents’ sense of security. J. Zhang et al. ^15^ proposed an automated urban safety perception assessment method integrating multimodal large language models with street-view imagery, providing an efficient automated tool for large-scale urban safety evaluations.

However, current research in the field exhibits certain limitations, primarily concentrating on data indicators of objective environmental characteristics while insufficiently addressing the spatial dynamics and variations among diverse populations. Furthermore, the psychological perceptions of spatial users have not been incorporated into the data scope. Although the utilization of multi-source big data has become increasingly prevalent, technical challenges persist in achieving comprehensive data integration, ensuring the interpretability of perception models, and guaranteeing the universality of applications^3,16^.

Urban safety research predominantly focuses on megacities and first-tier cities, with a notable lack of comparative studies across different urban types and regions. For instance, Fang et al.^17^ conducted research on urban safety perception in Shanghai, Qin et al.^5^ examined the same in Nanjing, and Wu et al.^18^ investigated the mechanisms influencing safety perception in Xiamen. Research on medium-sized and historic cities remains underdeveloped, and these gaps in urban safety perception research limit the universal applicability of the theory and policy guidance.

In summary, with technological advancements and theoretical deepening, urban safety perception research is evolving toward a multidimensional, dynamic, and intelligent approach. This study builds upon prior theoretical case studies of multisource urban data influencing safety perception. Utilizing the SHAP machine learning model and integrating individual psychological perception data, this study analyzes the nonlinear relationships and interactive effects between the built environment and psychological perception factors on urban residents’ safety perception. By examining the influencing factors of urban safety perception in Nanchang, a representative medium-sized city characterized by historical and cultural attributes, this study addresses previous research gaps, namely, the exclusion of psychological perception data and the scarcity of case studies on medium-sized cities. This study provides theoretical and data-driven support for developing a more scientific, precise, and resident-centric urban safety perception system, thereby offering a robust theoretical foundation and practical guidance for urban governance.

## Related works

### Definition and quantification of urban safety perception

Urban perception pertains to the psychological responses of residents to the visual environment of a city, serving as a pivotal research perspective for examining the relationship between individuals and the urban environment^19,20^. The concept of perceived safety, which first emerged in Freud’s psychoanalytic theoretical research, involves the anticipation of potential physical or psychological dangers or risks, along with an individual’s perceived capacity or incapacity to respond and manage them. It primarily manifests as a sense of certainty and control. In the context of urban planning, safety perception primarily reflects the complex emotional experiences and psychological sensations that arise when individuals within an urban environment synthesize potential physical and social environmental risks and other factors within their personal judgments^21–23^. Urban safety perception represents residents’ subjective psychological responses to potential risks within the urban environment, with its core manifestation being individuals’ emotional experiences regarding the “certainty” and “controllability” of urban space^22^.

In the early 20th century, international academia began focusing on the “sense of security” within urban spaces, proposing theories to enhance residents’ subjective safety experiences through urban planning, crime prevention, and environmental design. Among these, Jacobs introduced the “eyes on the street” theory in The Death and Life of Great American Cities^7^, emphasizing the importance of street vitality and diversity in enhancing security perceptions. Q. Wilson in Broken Windows^8^ demonstrated that environmental cleanliness and order significantly influence residents’ sense of safety. In recent years, advancements in information technology and big data have provided urban studies with more comprehensive data and perspectives on urbanization. Scholars have conducted in-depth quantitative research on urban safety perceptions across domains, including environmental perception^23^, infrastructure recognition, changes in social governance^24^, and shifts in economic levels.

### The influence of multi-source urban data on urban safety perception

In initial investigations, traditional quantitative approaches to assessing environmental perception predominantly utilized questionnaires and open-ended interviews. ^25^Through an analysis of surveys conducted across various European and American cities, Newman et al. ^27^ verified that constraints in time resources and significant labor costs posed challenges in acquiring extensive data samples. The deterioration of spatial environments and increased social disorder intensify residents’ feelings of unease. As urban research has progressed, scholars have identified that the mechanisms shaping perceptions of urban safety demonstrate multi-source heterogeneity^28^. Zeng et al. ^12^ observed that perceptions of urban safety arise from the interplay of variables such as the built environment, social factors, and individual activities, with the built environment being particularly influential. Consequently, the recent expansion of multi-source big data in urban areas has facilitated a more comprehensive and efficient collection of urban data from multiple dimensions for research on perceptions of urban safety. Among the aforementioned multi-source data factors, green coverage^11^, street enclosure^10^, and lighting facilities^8^ have been identified as key positive contributors, with these elements of the built environment significantly enhancing the perception of urban safety^29,30^. In terms of socioeconomic factors, Zeng et al. ^12^ noted that GDP density and housing prices indirectly bolster safety perceptions by enhancing regional governance capabilities. Fang et al.^17^ highlighted that affluent areas may lead to spatial segregation. Utilizing Nanjing’s urban districts as a case study, Qin et al.^5^ employed multi-source big data to investigate the intrinsic relationships among safety perceptions, the built environment, and the social environment. They also conducted a thorough analysis of the levels and distribution of safety perceptions at the urban scale, establishing an evaluation index system for urban safety perceptions.

Given that previous research has predominantly focused on megacities and first-tier cities, studies examining medium-sized cities—a more prevalent urban sample—remain insufficient^31,32^. This paper addresses this gap by selecting Nanchang, China, as a representative medium-sized city. Drawing upon its unique dual character as both a historic cultural city and a rapidly developing modern metropolis, it explores the factors influencing urban safety within this context.

### Study on factors influencing urban safety perception based on machine learning models

The advent of machine learning technology has fundamentally transformed the modeling paradigm of urban safety perception. In earlier investigations into the relationship between urban safety perception and its influencing factors, researchers predominantly utilized linear regression models to quantify and elucidate the impact mechanisms of key variables. Y. Zhang et al. ^9^ employed a multilayer linear model to demonstrate that high-density, mixed-use, and dense road networks within the built environment significantly diminish residents’ sense of safety. This adverse effect can be reversed into a positive protective mechanism, termed the “street eye,” in social environments characterized by strong community cohesion and high population homogeneity. Linear regression models, however, encounter limitations in the comprehensive analysis of multi-source urban data, including inadequate consideration of nonlinear relationships and limited causal inference capabilities. Recent advancements in machine learning and interpretable methods have facilitated a more profound exploration of the relationships between variables and urban safety perception through diverse machine learning approaches.

Among these diverse machine learning approaches, Liu et al.^33^ employed fully convolutional neural networks to segment street-view images into urban feature types, integrating perception scoring data with random forest algorithms to develop a six-category urban perception model for Wuhan’s urban environment. The SHAP interpretation method proposed by S. Lundberg & Lee^3^ unifies global and local interpretability, yielding results consistent with human perception. This effectively enhances the interpretability of machine learning and has been widely adopted across numerous fields. ^33^ Li et al.^14^ employed the SHAP machine learning framework to interpret and analyze environmental safety perception factors extracted from street view images, providing crucial support for improving urban traffic environments and enhancing pedestrian safety perception.

Previous studies primarily relied on multi-source data comprising physical and objective data from the built environment and social governance, inadequately incorporating individual psychological perceptions with environmental data into urban perception research. Consequently, this study integrates individual psychological perception data—including vitality, wealth, safety, and oppression perceptions—into multi-source data applications to explore the factors influencing urban safety perception.

## Methods and datasets

### Research framework

**Fig. 1:**
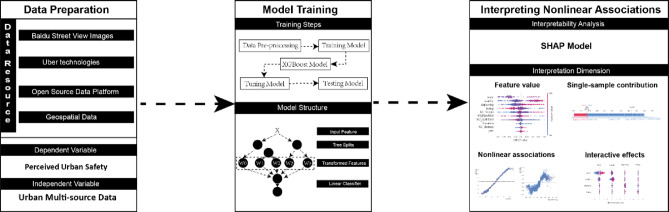
Methodology framework.

The objective of this methodological framework is to examine the diverse effects of multisource urban data on perceptions of urban safety. The proposed methodology consists of four steps: (1) perform a preliminary analysis utilizing the acquired multisource urban data within the designated study area and construct an analytical model accordingly; (2) model the relationship between multisource data indicators and perceptions of urban safety using the XGBoost model; (3) interpret the XGBoost model through the SHAP model to identify nonlinear global and local effects of multisource data indicators on various types of urban safety perceptions, along with key element interactions. Further analysis and corresponding planning recommendations are presented in the Conclusions and Discussion sections. Figure 1 illustrates the overall methodology framework of the article.

### Overview of the scope of the study

This study focuses on Nanchang City as the research area. As the provincial capital of Jiangxi Province, Nanchang demonstrates a moderate scale and pace of economic development relative to other economically advanced cities in China. Previous research on urban safety has often overlooked medium-sized cities. However, such cities, including Nanchang, play a crucial role in national development across various dimensions, such as the number of cities, their scale, and population size.

As a representative sample of a medium-sized city, Nanchang embodies the dual characteristics of a historic cultural city and a rapidly developing modern metropolis, making it highly illustrative of China’s urban development trajectory.

By selecting inner-ring urban data within Nanchang, the hexagonal hierarchical geospatial indexing system (hexagon side length of 0.1 km) provided by Uber Technologies was employed as the research unit, comprising 11,943 units. Compared to traditional point and line data, the hexagonal shape accounts for the spatial heterogeneity and coverage characteristics of street-view data^34^.

### Datasets and variables

#### Dependent variable: perceived urban safety

Perception data represent subjective interpretations formed by integrating the impact of various objective conditions on residents’ emotions into the foundation of raw urban spatial data.

In this study, urban safety perception data were acquired by utilizing Baidu Maps to generate sampling points at 50-meter intervals along road networks, totaling 37,924 points. Each point captures images in four directions (0°, 90°, 180°, and 270°) between 2018 and 2022. The analysis subsequently employed a deep learning model trained on the Place Pulse 2.0. Place Pulse 2.0, a dataset comprising 110,988 images from 56 cities across 28 countries, was developed by MIT’s Sense Lab. It quantifies human perceptions of urban appearance from a safety perspective, based on volunteer-tagged data. Ultimately, the trained model was applied to score the street-level imagery within Nanchang’s Third Ring Road, yielding the corresponding safety perception metrics.

#### Independent variables: urban Multi-source data

**Data Collection**.

**Fig. 2:**
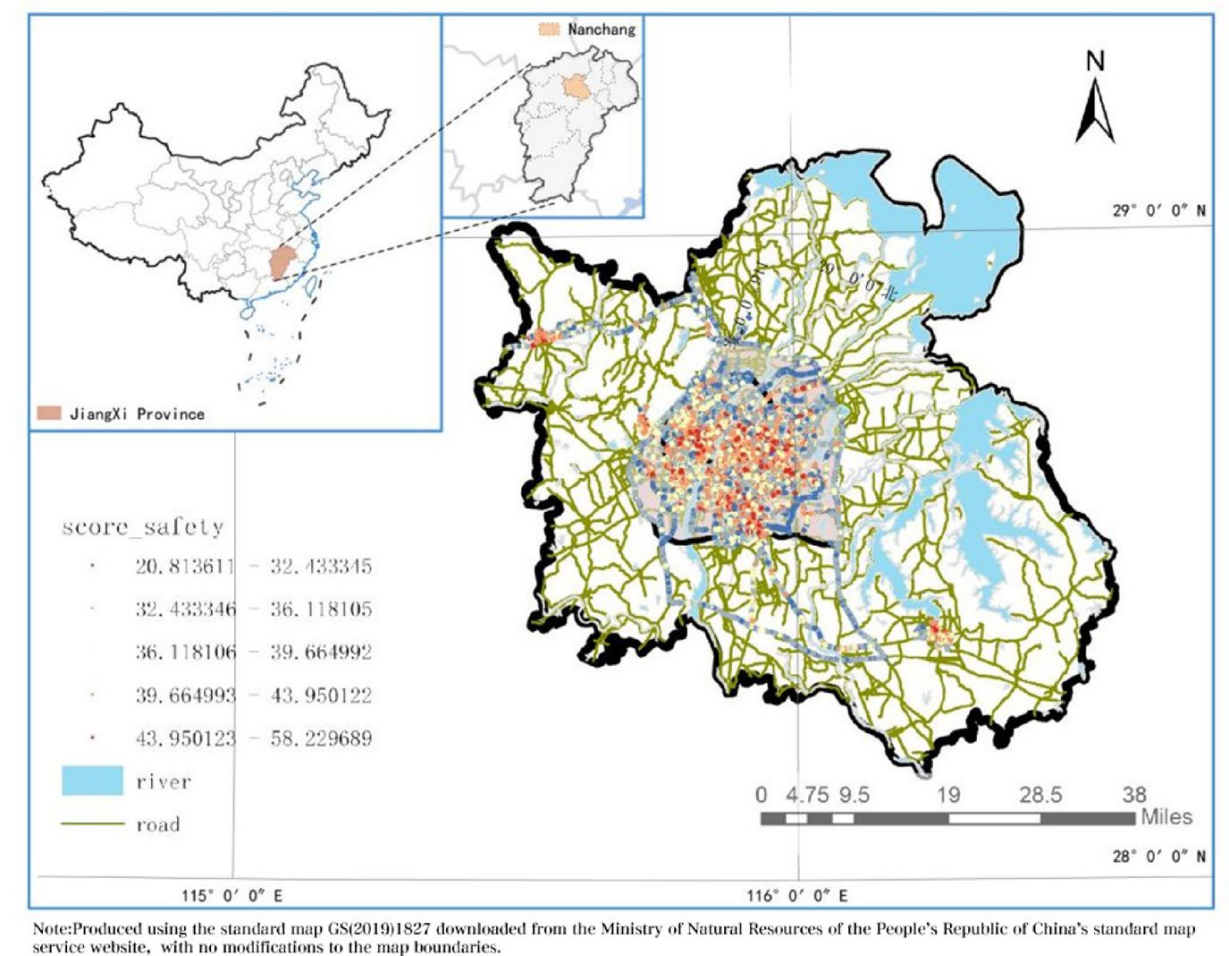
location analysis map.

The primary data sources for this study comprised the following multi-source urban datasets: (1) Vitality perception, wealth perception, and other perception data were derived from sampling point model scores; (2) 2022 night-time illumination data originated from VIIRS Nighttime Light^35^; (3) Points of interest data were sourced from the Baidu Maps Open Platform; (4) Elevation and gradient data were obtained from ASTER GDEM 30 M resolution digital elevation data via the Geospatial Data Cloud Platform; (5) 2022 NDVI data were sourced from the China 30-metre annual maximum NDVI dataset on the National Science and Technology Resource Sharing Service Platform; (6) Areas of interest for parks, green spaces, and water bodies were sourced from the Baidu Maps Open Platform; (7) 2022 housing price data were sourced from Lianjia; (8) 2019 population density data were sourced from the WorldPop database^36^; (9) 2019 GDP density data were sourced from the Resource and Environmental Science Data Platform; (9) Building height and silhouette data were sourced from the Geographical Remote Sensing Ecological Network Platform; (10) Road network data were sourced from the OpenStreetMap (OSM) platform; (11) Visual factors were extracted via the DeepLab V3 + model trained on the Cityscape dataset, calculating visual features per image and deriving ratios such as green view ratio and openness through formulae^37^; Fig. 2 illustrates the specific spatial distribution of the data and the representation of safety perception data within ArcGIS.

**Data Classification**.

**Fig. 3:**
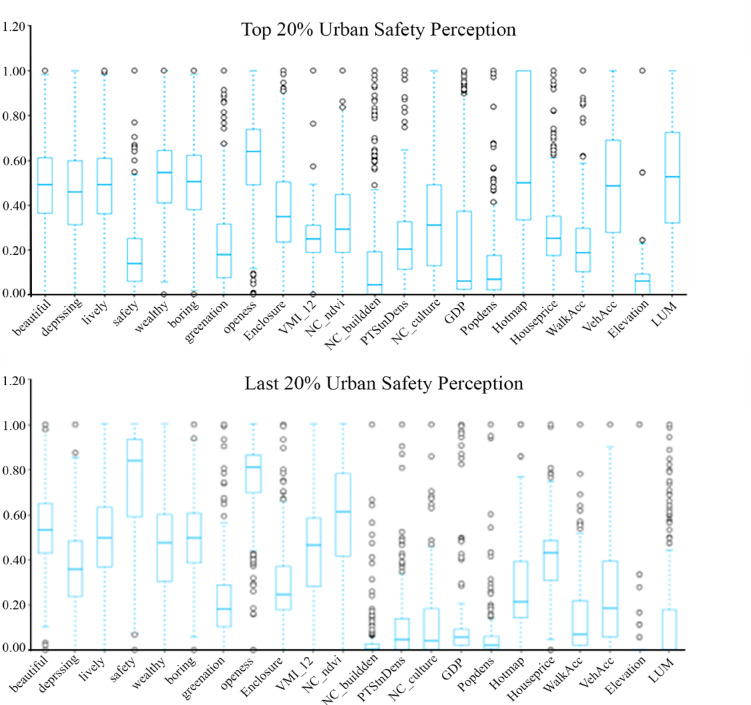
Differences in Urban Multi-source Data Indicators (Top 20% vs. Last 20%).

This study delineates its research boundaries utilizing urban dynamic perception data^38^, specifically LBS positioning heatmaps, and confines the sample space to areas exhibiting high population activity within the main urban district of Nanchang, particularly the region encircled by the Third Ring Road. Building upon the classical 5D theory framework of the built environment^39,40^, a hierarchically embedded measurement framework was developed. At the macro-level dimension, built environment elements were rigorously defined according to the 5D paradigm, establishing five constructs: Density (floor area ratio, building density), Diversity (land use mix entropy), Design (street network connectivity, cross-sectional width-to-height ratio), Destination Accessibility (POI accessibility index), and Distance to Transit (rail transit station coverage radius). At the meso-indicator level, 14 metrics were derived through spatial syntax (Depthmap), network analysis (ArcGIS OD Cost Matrix), and street-level semantic segmentation (DeepLabV3+) techniques. This indicator system transcends the two-dimensional limitations of traditional built environment measurements by integrating a three-dimensional evaluation framework that encompasses spatial topology (alpha index, integration), functional configuration efficiency (land-use mix index), and visual perception quality (green view ratio, sense of enclosure). This approach achieves a systematic representation of the multiscale built environment elements. Table 1 described a detailed description of the independent variables, and Fig. 3 illustrates the differences in urban mult-source data sources.

**Table 1:** Description of independent variables.

Variables	Description	Mean	Std. Dev.
Elevation (EL)	Distance from a point in the direction of the plumb line to the absolute base plane	5.409	11.250
Normalised Difference Vegetation Index (NDVI) Public Transport Station Density *(PubTransStnDens)* Walking accessibility *(WalkAcc)* Vehicular accessibility *(VehAcc)* Land use mix *(LUM)* Population density *(PopDens)* Housing price *(HousingPrice)*	One of the key parameters reflecting crop growth and nutritional information Kernel density of public transport stations $\:\mathrm{N}\mathrm{Q}\mathrm{P}\mathrm{D}\mathrm{A}=\frac{1}{\left|{\mathrm{R}}_{\mathrm{x}}\right|}\sum\:_{\mathrm{y}\in\:{\mathrm{R}}_{\mathrm{x}}}\frac{\mathrm{W}\left(\mathrm{y}\right)}{\mathrm{d}\left(\mathrm{x},\mathrm{y}\right)}$ $\:\mathrm{N}\mathrm{Q}\mathrm{P}\mathrm{D}\mathrm{A}=\frac{1}{\left|{\mathrm{R}}_{\mathrm{x}}\right|}\sum\:_{\mathrm{y}\in\:{\mathrm{R}}_{\mathrm{x}}}\frac{\mathrm{W}\left(\mathrm{y}\right)}{\mathrm{d}\left(\mathrm{x},\mathrm{y}\right)}$ SHDI Shannon Diversity Index for Land Use Population index for 1 km grid size Average house price in the region	4378.388 0.013 0.299 9.088 0.523 6596.778 11,094.943	1976.084 0.010 0.285 5.596 0.499 10333.430 4200.670
GDP density *(GDPDens)*	GDP index for 1 km grid size	37,594.420	56,555.859
Wealth Beauty Lively	Positive perceived score	43.634 42.651 35.740	6.885 9.455 8.035
Boring, depression	Negative perceived score	59.955 51.404	3.851 5.764

### Modelling approach

#### XGBoost model

XGBoost (Extreme Gradient Boosting) is an optimized distributed machine learning algorithm based on the gradient boosting framework. Its core mechanism achieves incremental model performance enhancement by integrating multiple weak learners (primarily decision trees)^1^. As an efficient engineering implementation of gradient boosting algorithms, this approach employs a strategy of iteratively correcting residuals from preceding models. This significantly enhances predictive accuracy while maintaining computational efficiency, leading to its widespread adoption in large-scale data modelling.

From an algorithmic architecture perspective, XGBoost employs forward stagewise additive modelling. Its prediction function can be expressed as a linear combination of K base learners:

$$\:\begin{array}{c}\widehat{{y}_{i}}={\sum\:}_{k=1}^{K}{f}_{k}\left({x}_{i}\right),\hspace{1em}{f}_{k}\in\:F\#（1）\end{array}$$

Where $\:{f}_{k}$ denotes the $\:k$th weak learner (decision tree), and $\:\mathcal{F}$ represents the hypothesis space. Given a dataset$\:\:D=\{\left({x}_{i},{y}_{i}\right){\}}_{i=1}^{n}$ comprising n samples and m-dimensional features, the model training process achieves parameter optimization by minimizing a regularized loss function:

$$\:\begin{array}{c}L={\sum\:}_{i=1}^{n}l\left({y}_{i},\widehat{{y}_{i}}\right)+{\sum\:}_{k=1}^{K}\varOmega\:\left({f}_{k}\right)\#（2）\end{array}$$

In the empirical analysis of this study, modelling was implemented using XGBoost 1.3.3 and Scikit-learn 0.24.2 within a Python 3.7 environment. The dataset was stratified into a training set (80%) and a test set (20%) according to stratified sampling principles. Hyperparameter optimization was performed using GridSearchCV combined with 5-fold cross-validation, focusing on tuning key parameters such as learning rate (η), maximum depth, and regularization coefficients (λ, γ). We have incorporated performance metric analysis using the test dataset into the original model code. Upon execution, the model performance metrics yielded MAE = 8.6827, RMSE = 10.9401, and R²=0.7226. These metrics indicate that the XGBoost model performs well. An R² value of 0.7226 suggests that, despite potentially containing a small number of extreme errors, the model captures the primary relationship between features and the target variable, demonstrating strong fitting capability. The relatively low and closely matched MAE and RMSE values indicate few extreme error samples, reflecting robust model predictions.

#### SHAP model

This study employs an interpretable machine learning framework grounded in cooperative game theory. Utilizing Shapley value decomposition methods, it achieves global feature attribution and local instance explanations for model predictions^3^, thereby revealing the nonlinear mechanisms through which multi-source heterogeneous data influence urban street vitality. The SHAP model, grounded in rigorous mathematical derivation, excels in satisfying axioms such as local accuracy, missing feature consistency, and linear consistency. It effectively handles nonlinear correlations and higher-order interactions within high-dimensional feature spaces.

According to Shapley value theory, the marginal contribution of the $\:i$th feature to the prediction result for instance x can be formally defined as:

$$\:\begin{array}{c}{{\upvarphi\:}}_{i}\left(x\right)={\sum\:}_{\mathcal{S}\subseteq\:\mathcal{F}\setminus\:\left\{i\right\}}\frac{\left|\mathcal{S}\right|!\left(\left|\mathcal{F}\right|-\left|\mathcal{S}\right|-1\right)!}{\left|\mathcal{F}\right|!}\left[{f}_{x}\left(\mathcal{S}\cup\:\left\{i\right\}\right)-{f}_{x}\left(\mathcal{S}\right)\right]\#（4）\end{array}$$

where$\:\:\boldsymbol{F}$ denotes the entire feature set,$\:\:\boldsymbol{S}$ represents the feature subset,$\:\:\mid\:\cdot\:\mid\:$ indicates the cardinality of the set, and $\:fx\text{}\left(\boldsymbol{S}\right)$ signifies the model’s conditional expected prediction value based on the subset$\:\:\boldsymbol{S}$. Consequently, an individual prediction can be decomposed into a linear combination of the baseline prediction and feature contributions:

$$\:\begin{array}{c}\widehat{y}\left(x\right)={{\upvarphi\:}}_{0}+{\sum\:}_{i=1}^{M}{{\upvarphi\:}}_{i}\left(x\right)\#（5）\end{array}$$

In the formula,$\:{\:{\upvarphi\:}}_{0}=E\left[f\left(x\right)\right]\mathrm{i}\mathrm{s}\:\mathrm{t}\mathrm{h}\mathrm{e}\:\mathrm{g}\mathrm{l}\mathrm{o}\mathrm{b}\mathrm{a}\mathrm{l}\:\mathrm{b}\mathrm{a}\mathrm{s}\mathrm{e}\mathrm{l}\mathrm{i}\mathrm{n}\mathrm{e}\:\mathrm{v}\mathrm{a}\mathrm{l}\mathrm{u}\mathrm{e}.，{{\upvarphi\:}}_{i}\left(x\right)\in\:R$ denotes the Shapley value for the i-th feature, where M represents the feature dimension. This additive feature attribution model achieves an organic integration of local fidelity in model prediction with global consistency in interpretation.

At the level of interaction effect analysis, the SHAP framework extends the definition to include the Shapley Interaction Index, capturing non-linear synergistic effects between features through second-order cooperative game decomposition. The interaction effect for feature pair (i, j) can be quantified as:

$$\:\begin{array}{c}{{\Phi\:}}_{i,j}\left(x\right)={\sum\:}_{\mathcal{S}\subseteq\:\mathcal{F}\setminus\:\{i,j\}}\frac{\left|\mathcal{S}\right|!\left(\left|\mathcal{F}\right|-\left|\mathcal{S}\right|-2\right)!}{2\left(\left|\mathcal{F}\right|-1\right)!}{\nabla\:}_{i,j}{f}_{x}\left(\mathcal{S}\right)\#（6）\end{array}$$

Where $\:{\nabla\:}_{i,j}{f}_{x}\left(\mathcal{S}\right)={f}_{x}\left(\mathcal{S}\cup\:\{i,j\}\right)-{f}_{x}\left(\mathcal{S}\cup\:\left\{i\right\}\right)-{f}_{x}\left(\mathcal{S}\cup\:\left\{j\right\}\right)+{f}_{x}\left(\mathcal{S}\right)$, this metric strictly satisfies the axioms of symmetry, zero-sum property, and additivity, enabling effective identification of super-additive (synergistic) or sub-additive (antagonistic) interaction patterns between features^41^ .

## Results

### Correlation check

**Fig. 4:**
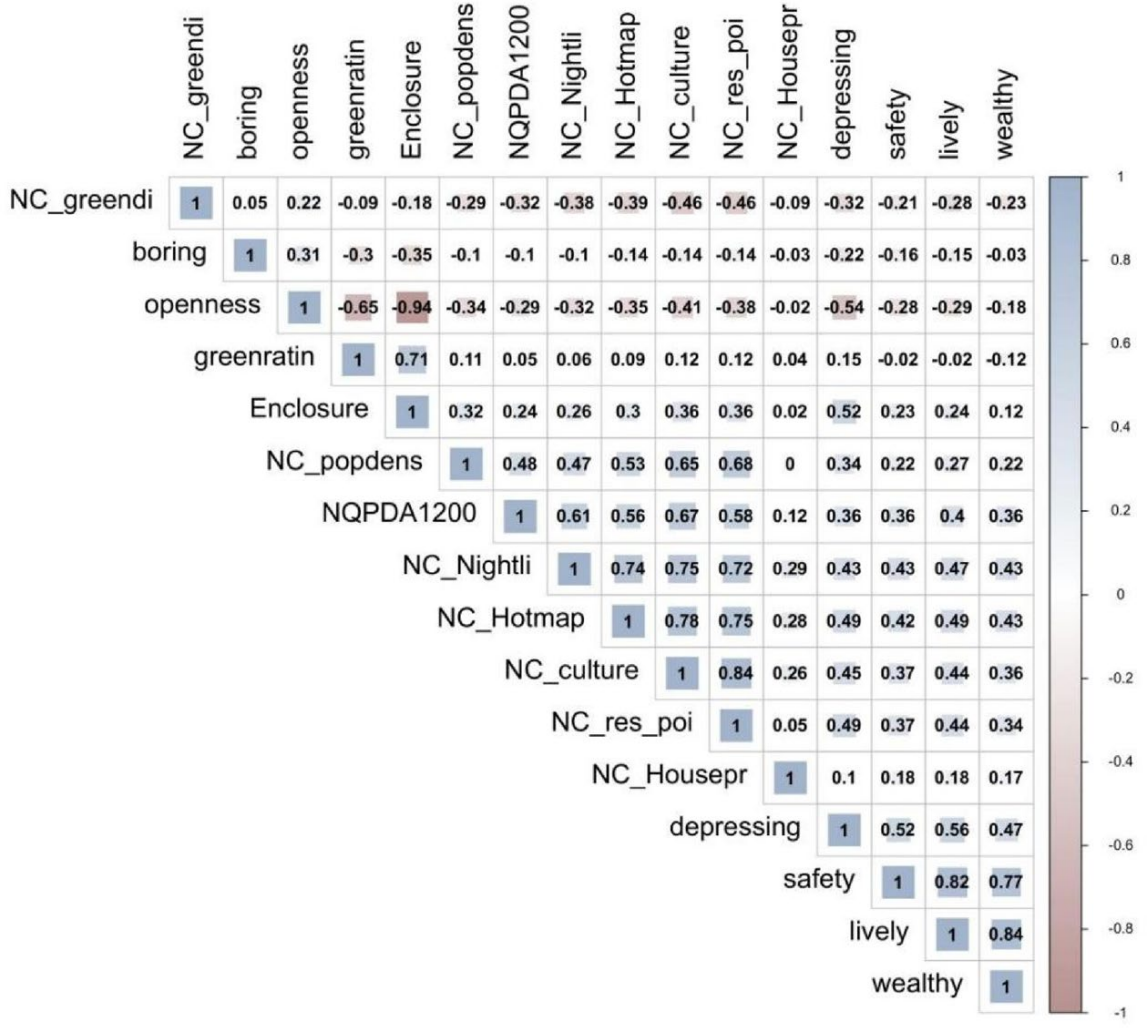
Pearson Correlation Analysis.

To optimize data preparation for model operations, preprocessing was conducted prior to model integration. This included regression analysis, multicollinearity assessment, and Pearson correlation analysis (Fig. 4. ), ensuring more accurate fitting outcomes and mitigating the influence of variables with low correlation coefficients on overall results. Table 2 illustrates evaluation of model fitting performance.

As shown in Tables 2 and 3, the R-squared value for the selected multi-source urban data and urban safety perception data is 0.718, indicating a high degree of data fit. Furthermore, the ANOVA variance analysis yielded an F-value of > 100, with a significance level below 0.01, confirming the data’s favorable fitting status. Further examination of the variance inflation factor (VIF) for multicollinearity revealed that while the VIF for HOSPITAL-POI was substantially above 10, warranting its direct exclusion, the VIFs for enclosure and culture were around 10 without significant multicollinearity traits. In past research, street enclosure has frequently emerged as a key factor in assessing perceptions of urban safety. For instance, in Jacobs’ The Death and Life of Great American Cities^7^, it was proposed that open, vibrant street interfaces enhance perceived safety by increasing pedestrian interaction. Zhang Yanji et al. ^9^similarly identified street enclosure and open space layout as key spatial parameters influencing residents’ safety perceptions. Concurrently, regarding cultural data impacts, Qin Xiao et al.^5^ utilizing multi-source big data, investigated the influence of urban cultural factors on residents’ safety perceptions, highlighting the role of cultural spaces and community attributes in safety perception. Based on a comprehensive review of prior research, it has been decided to provisionally retain these two factors. Further research will integrate additional variables before determining whether data reduction is warranted. The specific test values are shown in Table 3. In the Pearson correlation analysis depicted in Fig. 4, after excluding indicators with indices approaching zero, the two variables exhibiting the highest positive correlations were lively (perceived urban vitality) and wealthy (perceived urban wealth). This suggests that urban vitality and urban wealth may be strongly correlated with perceptions of urban safety.

**Fig. 5:**
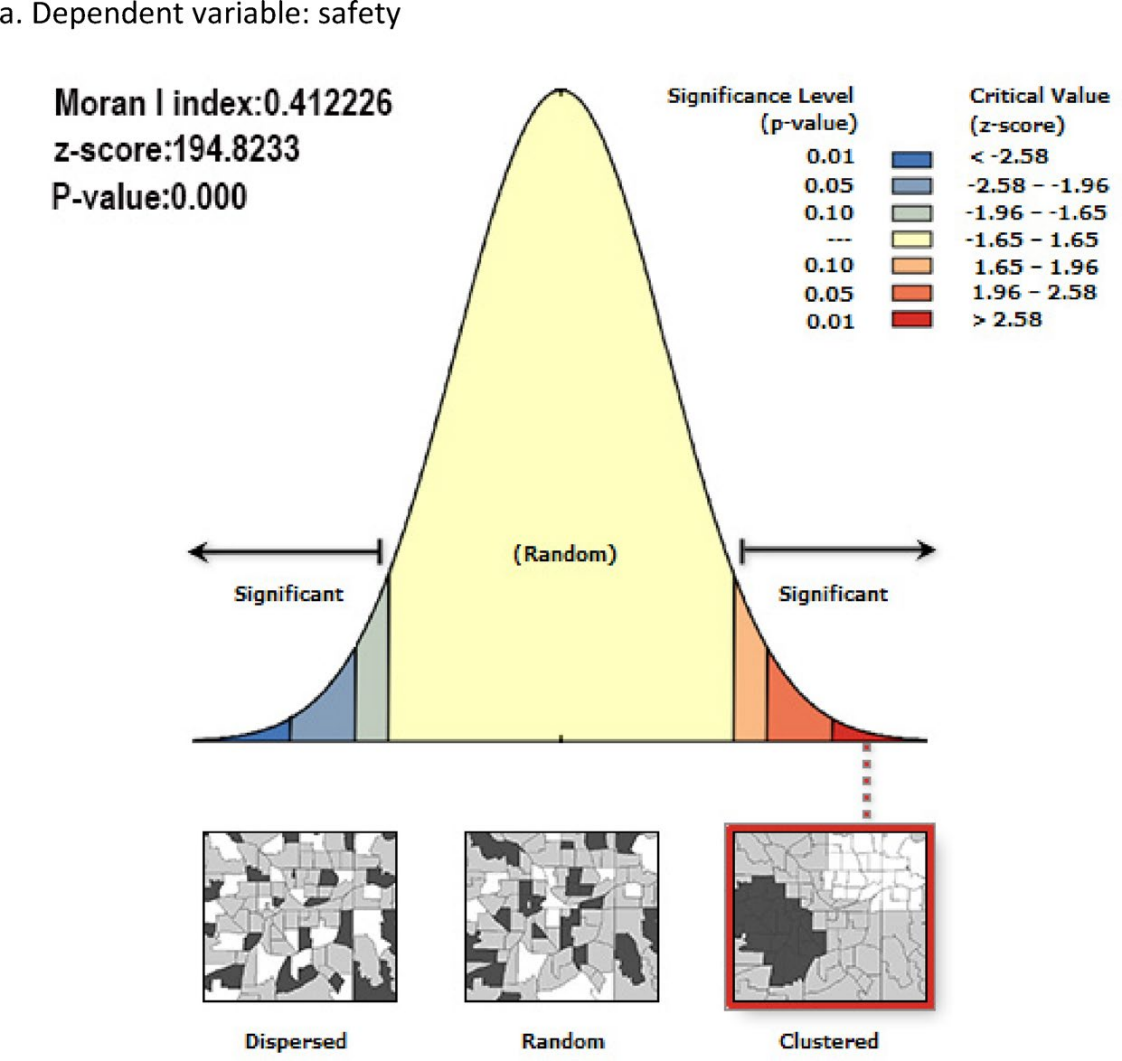
spatial autocorrelation processing method.

Finally, the study employed the Moran’s I method to examine spatial autocorrelation. The results indicate that Moran’s I = 0.412226, with a p-value approaching zero. This index suggests a moderately high positive spatial autocorrelation value, indicating a pronounced spatial clustering phenomenon among variables within the study area. That is, spatial units exhibit a high degree of similarity in characteristics, and the data distribution is not random. This spatial autocorrelation analysis demonstrates that the urban indicators under investigation exhibit significant spatial clustering characteristics. Figure 5 illustrates the specific numerical performance of the experiment.

**Table 2:** Evaluation of model fitting Performance.

Model Summary ^b^				
Model	R	R	Adjusted R-squared	Standardised Estimation Error
1	.847^a^	0.718	0.712	2.568622105389

**Table 3:** VIF multicollvmiinearity Coefficients.

Independent variables	Multicollinearity statistics (VIF)
Beautiful	2.346
Depressing	3.302
Lively	4.188
Wealthy	3.817
Dull	1.234
Greenrating	2.854
Openness	8.866
Enclosure	10.489
NC_ndvi202	1.529
Greendi	1.513
NC_Riverdi	1.384
NC_station	3.384
NC_subway	3.826
NC_roadden	2.940
NC_res_poi	7.573
NC_Nightly	3.636
NC_popdens	3.028
NC_Hotmap	3.725
NC_Housepr	1.938
NQPDA9500	2.305
SHDI	2.023
Elevation	1.277

### Non-linear interpretation

#### Feature importance of urban Multi-source data across dimensions

Table 4 presents the global feature importance values for various multi-source urban data indicators influencing perceived urban safety under the XGBOOST model. Variables are ranked by global importance from highest to lowest, displaying the top 10 most influential features across each variable metric. Among these, indicators with relatively high feature importance include perceived urban vitality, perceived urban wealth, and perceived oppression.

Specifically, within the spatial multi-source data, the perception of urban vitality proves most crucial in influencing perceptions of urban safety, exhibiting significantly higher feature importance than other indicators. Additionally, the perception of urban wealth demonstrates considerable importance for urban safety perceptions. The remaining indicators within the top ten exhibit broadly comparable levels of importance, though these remain relatively low.

**Table 4:** Ranking of feature importance for perceived urban safety from Multi-source urban Data.

Feature	Importance
Lively	0.49544
Wealthy	0.11943
Depressing	0.02165
NQPDA9500	0.02143
NC_subway	0.01749
NC_Nightly	0.01677
boring	0.01305
NC_educate	0.01303
NC_culture	0.01295
NC_NDVI202	0.01286

**Fig. 6:**
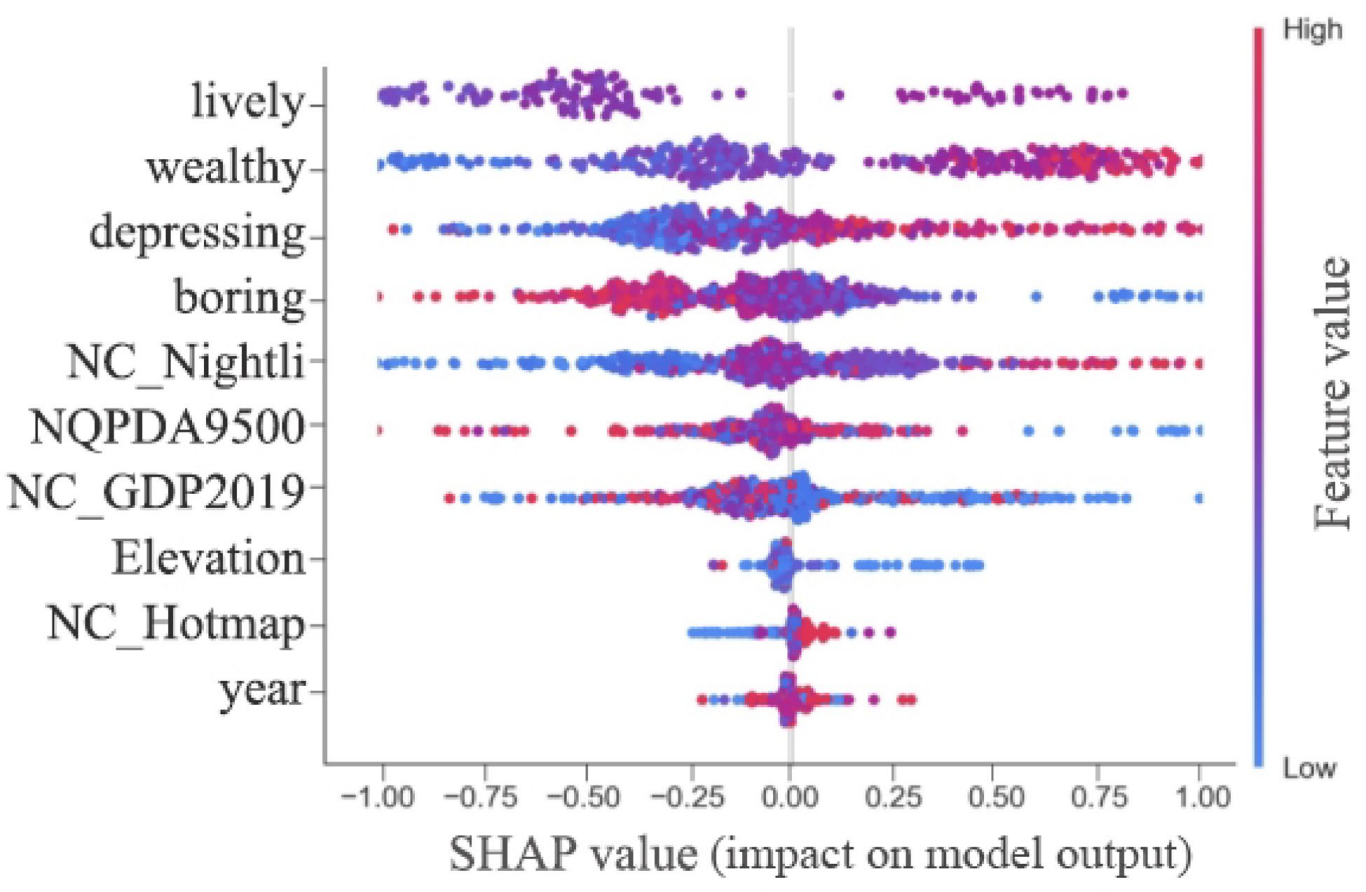
Ranking of SHAP value contributions from multi-source urban data to perceived urban safety.

As illustrated in Fig. 6, this depicts the overall impact of each indicator’s contribution value on urban safety perception. By incorporating SHAP operations onto the XGBOOST model, the contribution values of each urban data element to urban safety perception were derived. Their positive or negative influence can be intuitively determined through data color and coordinate placement.

Figure 6 reveals that urban vitality perception, the most influential factor, exhibits a relatively polarized SHAP value distribution. This indicates that urban vitality perception significantly impacts urban safety perception in both positive and negative directions. Comparatively, points representing positive influence tend towards redder hues than those indicating negative influence. That is to say, whilst the perception of urban vitality exerts substantial bidirectional influence on urban safety perception, its positive impact contributes more significantly. Concurrently, higher levels of urban wealth yield a greater positive contribution to urban safety perception, consequently elevating the perceived level of safety. Furthermore, increased levels of oppression exert a certain promotional effect on urban safety perception, whereas heightened levels of tedium and urban road network integration exert a negative influence. Factors such as urban GDP, building height, traffic density, and year of survey do not exert significant influence.

#### Non-linear relationship between multi-source data and urban safety perception

**Fig. 7:**
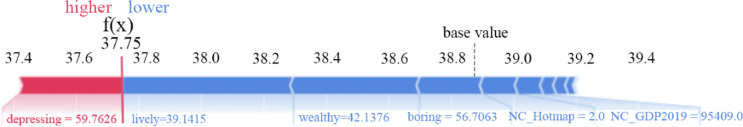
Single-sample SHAP feature contribution plot.

Single-sample feature plots (Fig. 7) provide a visual representation of the contribution of individual samples to the SHAP model outputs. Each feature value of a sample point acts as a factor that either positively or negatively influences its overall contribution. The contribution prediction begins from a baseline, which represents the mean model output across all sample points, denoted as the fundamental average f(x) value. Each sample attribution is depicted by an arrow, indicating its contribution to the prediction outcome. Blue indicates a feature exerting a negative influence on the prediction, while red signifies a feature with a positive influence. This particular sample point represents a randomly selected sample of 115/17,010. As illustrated in Fig. 7, the baseline mean f(x) for all sample points was approximately 38.91, whereas the f(x) value for this sample point was 37.75. In terms of overall characteristics, it exerts a negative influence on the comprehensive safety perception model. Specifically, aside from feature suppression, which exerts a positive influence on the overall model, other features, such as vitality perception and wealth perception, exert negative effects. Among these, the urban GDP factor had the most significant negative impact.

Global feature importance (Table 4) and the overall impact of multi-source urban data elements on city safety perception (Fig. 6) indicate that urban vitality perception, urban wealth perception, and suppression degree exert the most significant contributions to city safety perception. Consequently, these three feature elements were selected for further analysis. Figure 6 illustrates how SHAP values for observed city safety perception change as feature element values vary.

**Fig. 8:**
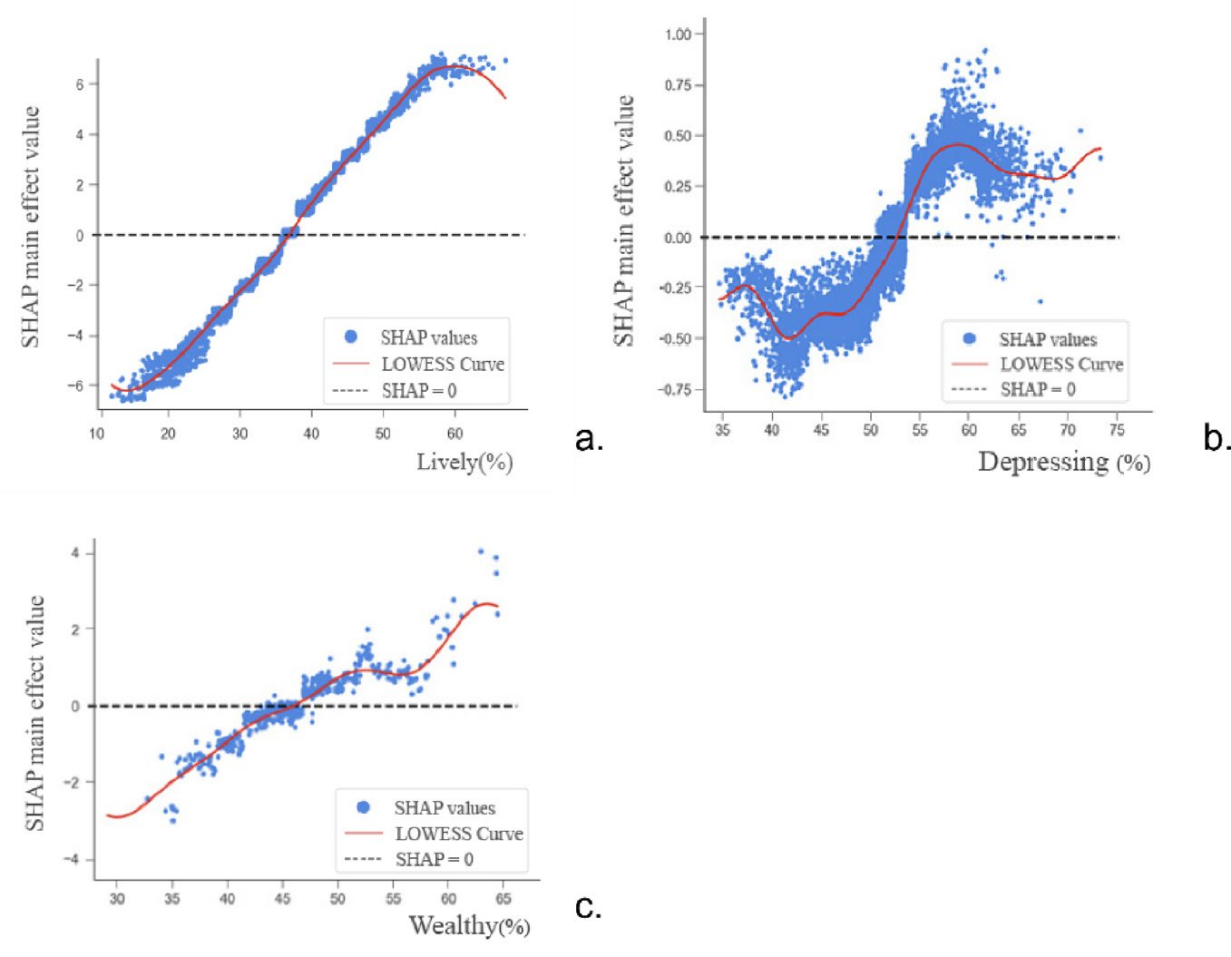
Relationship between multi-source urban data and their SHAP values (perceived urban lively, wealthy, depressing).

The SHAP value for the perceived urban vitality feature(Figure 8)exhibits an approximately linear increasing relationship with perceived urban safety. Enhanced perceived urban vitality implies more frequent population activity, corresponding to higher levels of economic and social development and urban construction standards, thereby elevating perceived urban safety. When the perceived urban vitality exceeds approximately 38%, the contribution of this feature to perceived urban safety shifts from a negative to a positive influence. However, beyond a certain threshold—observed in the figure to be around 60%—the positive contribution to perceived safety begins to diminish. This indicates that when urban activity becomes excessively vigorous, surpassing the threshold of the city’s management capacity, safety risks increase accordingly, leading to a situation where the positive impact becomes limited.

Figure 8 illustrates the relationship between perceived urban wealth and perceived urban safety, showing an approximately linear increasing trend with data fluctuations. Within the 30%-53% range of perceived wealth, higher wealth perceptions consistently elevate the SHAP value for safety perception. Between 53% and 58%, the positive impact on safety perception diminishes despite rising wealth perceptions. Beyond 58% perceived wealth, the SHAP value resumes an upward trajectory. This shift may indicate that heightened perceived wealth generally enhances urban safety perceptions by elevating overall urban development levels. However, as urban wealth growth simultaneously generates more negative social and economic issues, it can impede further positive gains in perceived safety once a certain development threshold is reached.

Figure 8 illustrates the more intricate relationship between perceived oppression and urban safety perception, showing an overall increasing trend amidst fluctuating patterns.

#### Interaction effects of key variables on perceived urban safety

**Fig. 9:**
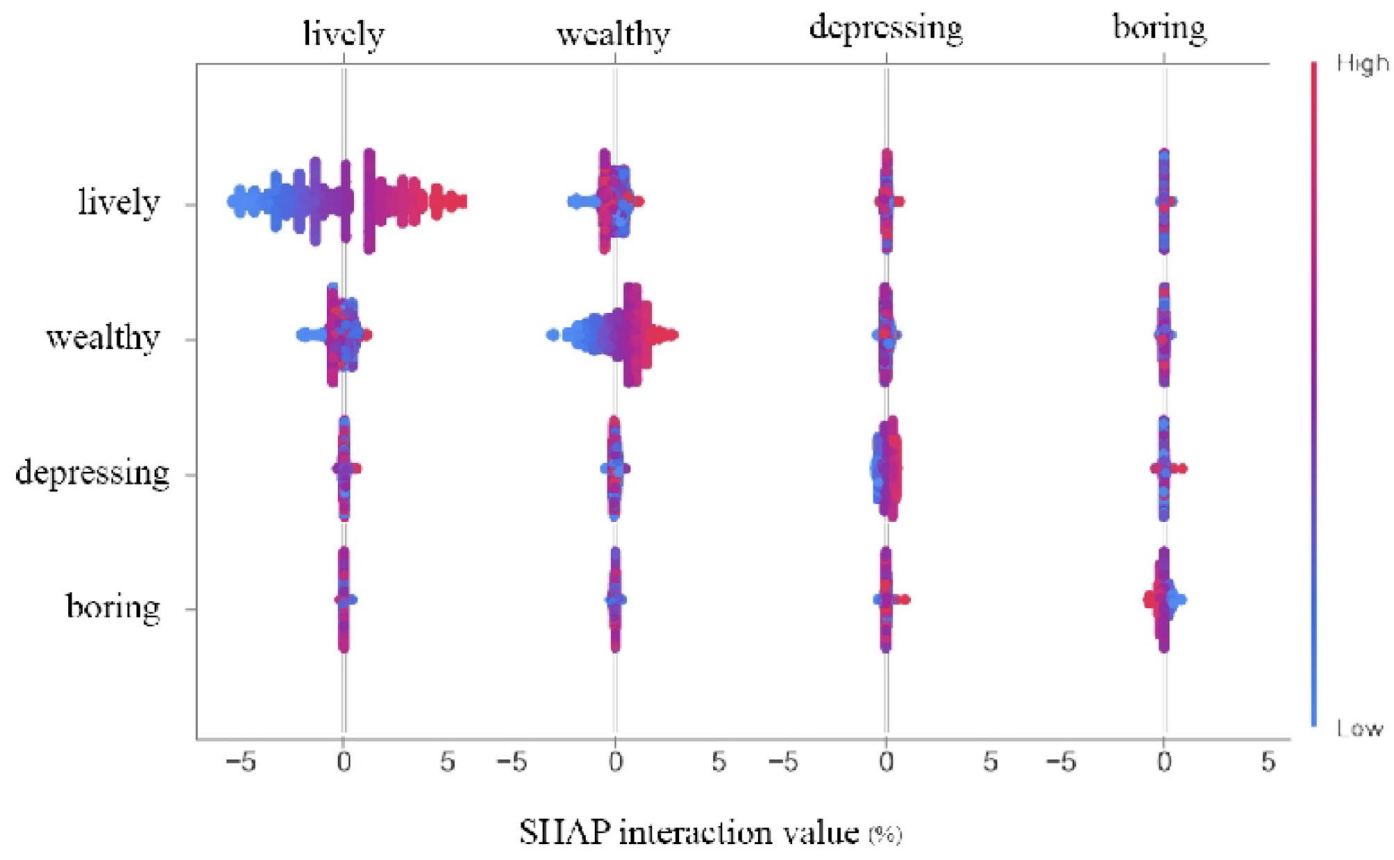
Dual-feature SHAP interaction effect.

Figure 9 Visualizes the bivariate interaction effect analysis among high-impact characteristics. Results indicate that, controlling for the independent main effects of each characteristic, a significant synergistic effect exists between perceived urban vitality and perceived urban wealth, with the highest interaction strength among the four characteristic groups. Further analysis reveals observable interaction effects between perceived oppression and perceived tedium, as well as between perceived vitality and perceived tedium.

**Fig. 10:**
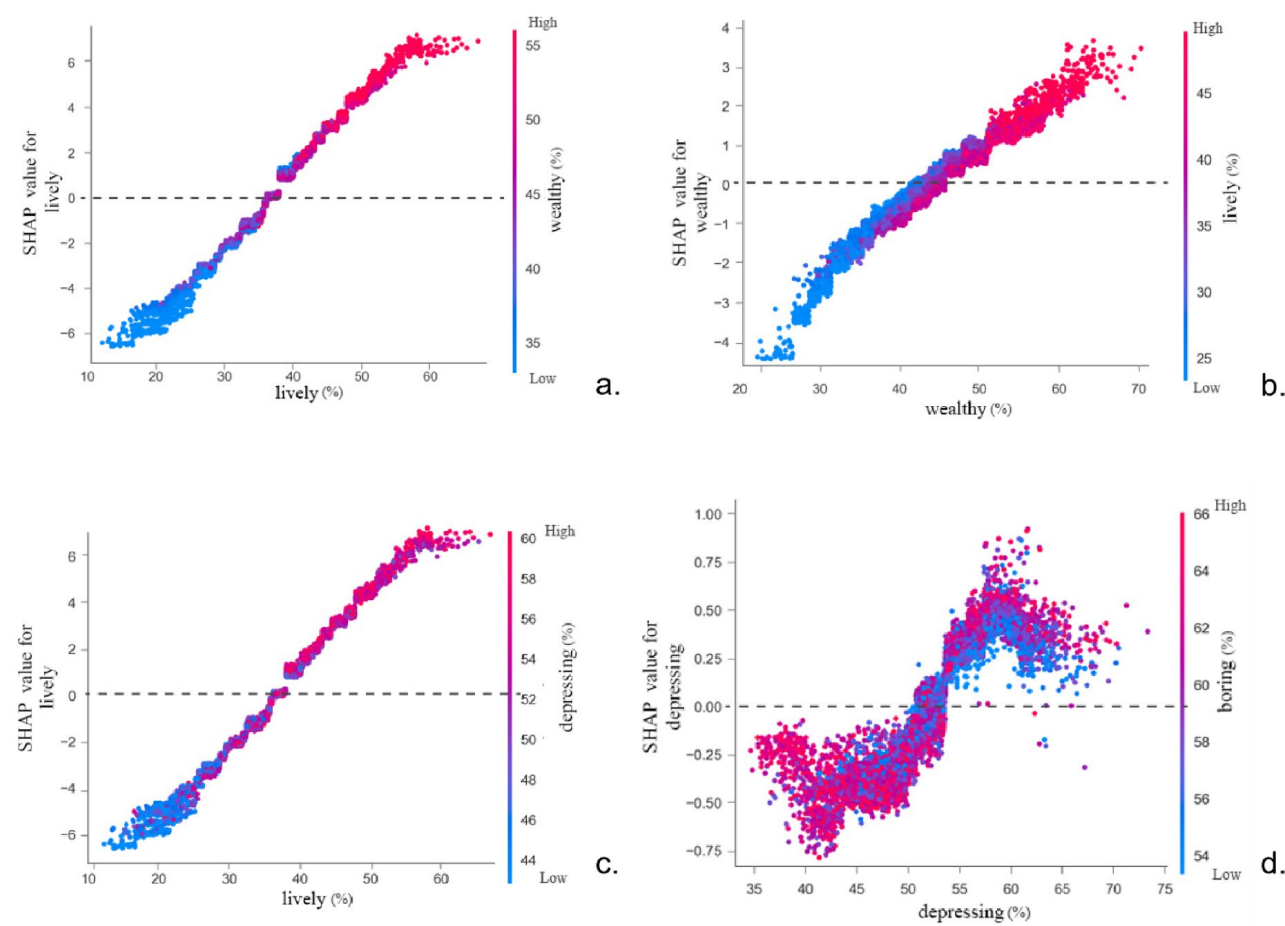
SHAP interaction effects between key variables.

Figure 10 presents the SHAP interaction plots for two feature variables. As shown in Fig. 10, the horizontal axis displays variations in perceived urban vitality, the right vertical axis shows changes in perceived wealth, and the left horizontal axis indicates SHAP values. When the perceived urban vitality falls below 40%, a significant number of blue points lie beneath the red points. This indicates that when perceived urban vitality is below 40%, an increase in perceived urban wealth diminishes the perceived urban safety. Similarly, when using the SHAP value of perceived urban wealth as the metric, as shown in Fig. 10, an increase in perceived urban vitality reduces perceived urban safety when perceived urban wealth is below 40%. The underlying reason is that when urban wealth perception is excessively low—indicating underdeveloped urban conditions—the city lacks sufficient economic resources and infrastructure to sustain safety maintenance and development. Consequently, when population vitality exceeds the city’s carrying capacity, the probability of criminal activity increases, alongside a higher likelihood of public safety incidents. This poses significant risks to urban security, thereby diminishing the sense of safety.

Figure 10 indicates that when perceived urban vitality exceeds 30%, higher levels of perceived oppression correlate with increased perceived urban safety. Figure 10 illustrates the interaction between perceived oppression and perceived tedium in relation to SHAP values.

Analyzing the data collectively reveals that perceived urban safety exhibits positive correlations with both perceived urban vitality and perceived urban wealth. These relationships are fundamentally linear and positive: when vitality and wealth perceptions are low, they exert a negative influence. However, once perceptions exceed a certain threshold, higher values yield greater positive impacts on perceived safety.

#### Analysis of key variables

In summary, the two key variables exerting the greatest influence on urban safety perception were the perceptions of urban vitality and urban wealth. The following section conducts an exploratory analysis of the key variables that significantly impact urban safety perception.

(1) Specifically, the data indicate that urban vitality perception, the variable with the greatest contribution to safety perception, is reflected through urban functionality and urban spatial interface indicators, which embody residents’ quality of life and the city’s high-quality development level. Thus, the vitality index reflects the diversity of human interactions within a region, and the impact of such diversity on urban safety perception should be measured according to regional development levels and the specific circumstances of physical street and interface indicators. The positive correlation between enhanced vitality indices and perceived safety is corroborated by Jacobs’s three principles of street safety, which emphasize that diverse street uses are essential for ensuring urban street security.

However, as illustrated in Fig. 8, when perceived urban wealth was low, perceived vitality was negatively correlated with perceived safety. Such areas may be found in older neighborhoods lacking unified planning, or in underdeveloped urban development zones.

In unrenovated historic districts where urban wealth levels remain relatively low, heightened vitality may introduce external populations, public activities, and projects without proper planning. This can lead to social issues such as complex demographic profiles and high population turnover. Within the security domain, this may result in increased crime rates, whereas in the traffic safety sphere, it can cause congestion, elevated accident probabilities, and other hazards. Concurrently, in older districts, where streets typically feature narrow, elongated layouts, increased population activity may disrupt spatial order and visually heighten pedestrians’ perceptions of insecurity.

For underdeveloped urban development zones, these areas feature sparse landmarks and open spaces. However, owing to their low level of development and perceived scarcity of wealth coupled with inadequate infrastructure and limited urban functions, the indiscriminate introduction of population vitality can degrade the spatial environment and disrupt the existing social structure. Consequently, this diminishes residents’ perceptions of urban safety. Although enhancing population vitality may improve street surveillance rates, within complex demographic networks characterized by unfamiliar interactions and a lack of interpersonal trust, overly open and transparent boundaries offer little benefit to individual safety perceptions. Such environments may exacerbate residents’ unease.

(2) Regarding wealth perception, which significantly influences urban safety perception, this indicator reflects residents’ evaluative judgement of a city’s affluence based on their experience with its interfaces and functions. Elevated levels primarily signify rising economic standards, and their impact on urban safety perceptions is explained through three distinct dimensions.

As illustrated in Figs. 9 and 10, the urban wealth perception exhibits a positive correlation with the urban safety perception once both surpass a low threshold (approximately 45%, as indicated in Fig. 10). Regarding activity safety, an enhanced wealth perception drives economic growth, which facilitates improvements in urban spatial interfaces and street environments. Infrastructure, such as footpaths, becomes more comprehensive and orderly, while spatial enclosure and green visibility rates gradually increase. Regarding traffic safety, urban road planning and traffic management enhance the perceptions of the transport environment. In terms of defense safety, elevated urban economic levels strengthen governance and infrastructure development, improve public security, and facilitate the construction of facilities, such as street lighting and barriers. This reduces crime rates and contributes to heightened perceptions of safety among residents.

However, when urban wealth perception develops to the point where motor vehicle numbers exceed the city’s carrying capacity, the density of buildings along streets becomes excessive, sky visibility diminishes, and green visibility rates decline during development, urban disorder ensues. At this stage, rising wealth perceptions may cause fluctuations or even declines in residents’ sense of urban safety. Similarly, enhancing perceived urban affluence when vitality is low, such as in densely built-up areas devoid of human activity, can diminish the sense of security of residents.

(3) Beyond the two primary contributors mentioned above, perceptions of urban safety also correlated with feelings of urban oppression and urban tedium. These can be broadly understood as indicators of urban interface permeability and functional diversity, the impact of which requires analysis tailored to specific neighborhoods.

Generally, increased oppressiveness signifies reduced interface permeability, an indicator of heightened building density and rising economic indices, and thus tends to correlate positively with perceived safety. Meanwhile, perceived urban tedium relates to functional diversity, a metric that manifests differently across distinct neighborhood types. For instance, in economically prosperous historic districts, enhanced functional diversity fosters greater ‘street eyes’. Residents can more readily monitor the multifunctional use of urban spaces in daily life, fostering a sense of a stable and reliable public order that cultivates security. Conversely, in sparsely populated urban development zones with high population mobility, where trust-based social relationships remain underdeveloped, increased functional diversity and street surveillance fail to enhance the perceived safety. Instead, mirroring the mechanism of heightened population vitality alone diminishes the residents’ sense of security.

To validate this hypothesis regarding historic districts, the study further selected the corresponding latitude and longitude data from Nanchang’s historic district for reduction. After the selected data were processed, the contribution values to urban safety were recalculated for the remaining data. The selection of historical conservation areas requires the simultaneous fulfilment of two criteria: the area must possess a long history and hold conservation value. Specific reference points for assessment are areas already designated as historical landscape zones under regulations such as the Regulations on the Protection of Historical and Cultural Cities, Towns and Villages, and the Nanchang Historical and Cultural City Conservation Plan. Concurrently, as the research prioritises urban residents’ safety perceptions, the delineated areas should emphasise residential living functions rather than protective heritage sites.

In summary, two historical districts—Tengwang Pavilion and Sanyanjing—were selected for data processing. The Tengwang Pavilion Historic Landscape Zone is bounded by Yangming Road to the north, Zhongshan West Road to the south, the Gan River to the west, and Zigou Road to the east, covering a total area of 81.57 hectares. Within this zone, the Tengwang Pavilion Scenic Area constitutes the core protected area spanning 4.45 hectares, with a construction control zone of 17.5 hectares. This district embodies the profound cultural heritage of the ancient Yu Zhang Commandery, bearing witness to the city’s evolution and development. It epitomises traditional Chinese architectural aesthetics, alongside courtyard-style community spaces and textures exemplified by the Provincial Anthem Artistic Quarter.

The Sanyanjing Historic District encompasses cultural landmarks such as the Yu Zhang Academy and the Wan’an Yunsheng Examination Hall; As a witness to pivotal historical legends of Nanchang, the Sanyanjing Historic District and its surrounding areas possess significant historical and cultural value, making its preservation critically important for Nanchang. It largely retains the historical appearance of Nanchang’s residential architecture from the Qing Dynasty to the Republican era, serving as a tangible testament to the city’s evolution. Its internally preserved street and alleyway fabric reflects a distinctive urban layout.

Following site selection, we delineated boundaries according to the government’s control detailed plan for historical districts. After obtaining precise latitude and longitude coordinates via Baidu Maps, we conducted area selection and data filtering within the raw dataset.

**Fig. 11:**
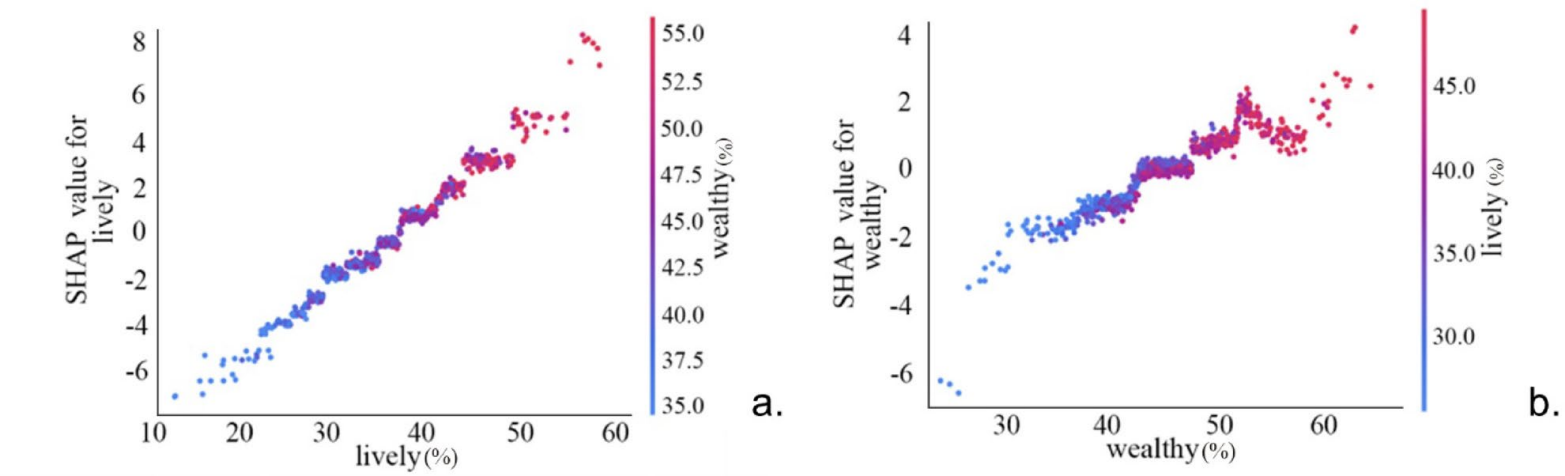
Interactive influence after data cleansing of the historic old town.

The results are shown in Fig. 11 Consistent with this hypothesis, the figure clearly demonstrates that after removing data from the old urban area, the reduction in both low and high positive contributions was most pronounced.

This simultaneously validates the earlier hypothesis within the visualized data: in older, historically developed urban districts lacking unified planning, where perceptions of urban wealth are low, vitality perceptions exhibit a negative correlation with perceptions of safety. Concurrently, owing to their longstanding development, population density, and frequent central urban locations, these areas also feature numerous zones with perceptions of high vitality. Consequently, a polarized development dynamic emerges within these districts, presenting a dilemma for urban management. Because of their unique historical and cultural significance, these areas must be managed in a manner that safeguards their traditional civic economy and historical character while adapting to contemporary urban governance trends. This dual approach ensures that they retain their core status, showcasing both the city’s heritage and evolving modern identity.

## Discussion

### Key findings

This study introduces multi-source urban data to construct XGBOOST and SHAP models, investigating the contribution of these data to perceptions of urban safety and how factors interact. This provides more specific guidance for urban planning. Results indicate that perceptions of urban safety are influenced non-linearly by multi-source data, with vitality and wealth perceptions showing the strongest correlations. The influence mechanism is moderated by the city’s development stage and spatial heterogeneity. The XGBoost model’s fit (R²=0.718) and variance test (F = 125.358, *p*<0.001) demonstrate high explanatory power and statistical significance. Feature importance analysis based on SHAP values further reveals that perceptions of urban vitality and wealth contribute most significantly to safety perception, exhibiting positive correlations but with threshold effects. When perceived vitality exceeds 40%, its positive impact on perceived safety significantly intensifies; conversely, in areas where perceived wealth falls below 45%, increased vitality may paradoxically diminish perceived safety. Furthermore, the contribution of perceived oppression to perceived safety exhibits complex fluctuations, potentially linked to regional heterogeneity in urban interface permeability and functional diversity.

The findings reveal that perceptions of urban vitality and affluence exert an absolutely relevant influence on perceptions of urban safety. They further demonstrate that perceptions of urban safety constitute a composite outcome shaped by the interactive effects of key variables, rather than being determined by the linear variation of any single factor. This aligns with the findings^9^ and supplements Jacobs’ theory of the ‘eyes on the street’, suggesting that the protective mechanism of the ‘eyes on the street’ can only function effectively within the concurrent influence of social vitality and socio-economic development. Compared to existing literature, this study’s integration of multi-source data analysis and machine deep learning provides a quantifiable, efficient, and objective evaluation method for urban safety perception. The findings support research by Fang et al.(2023)and Qin et al. (2024), confirming that multi-source urban data can contribute to perceptions of urban safety. This research offers a comprehensive explanation grounded in machine learning model data computation for enhancing residents’ perceived safety within urban planning. By applying the model to Nanchang City as the study area, it fills a gap in previous urban safety research concerning medium-sized cities, providing more comprehensive and detailed evidence for studies on urban safety perception.

The study reveals that key variables such as perceptions of urban vitality and wealth exhibit nonlinear relationships and threshold effects with urban safety perceptions. Specifically, the influence of these variables on safety perceptions undergoes significant shifts once they surpass certain thresholds. This finding complements the conclusions of Qin et al.(2024), providing attribution support for the need to consider multiple dimensions in urban safety optimization strategies. This research contributes novel theoretical data to urban safety perception studies, indicating that future urban planning and development strategies must fully account for the nonlinear relationships and threshold effects among various factors. This approach avoids the pitfall of blindly pursuing improvements in certain indicators while overlooking their potential adverse consequences.

Unlike existing urban safety perception studies that predominantly rely on street imagery for quantifying street safety through image semantic analysis, this research innovatively incorporates the contribution of diverse psychological perception data to safety perception. Building upon the traditional 5D theory of the built environment, it incorporates multidimensional psychological perception data from pedestrians—such as perceptions of vitality, affluence, and oppression—to assess their impact on safety perception. This integration of environmental psychology establishes a comprehensive evaluation framework combining multi-scale built environment elements with psychological perception interventions. This theoretical innovation enables comprehensive multi-dimensional assessment of factors influencing urban safety perception, addressing existing research gaps in pedestrian psychological perception and overcoming the limitations of single-dimensional analysis.

Based on the aforementioned analysis of multi-source urban data, this paper proposes corresponding renovation recommendations for urban planning and urban renewal, categorized under the three major domains of safety perception. Regarding activity safety, to enhance the diversity and accessibility of public spaces, more parks, squares, and pedestrian streets should be constructed. This would promote social interaction and activity participation among residents. Concurrently, mixed-use development should be encouraged by incorporating land-use integration into urban planning, combining commercial, residential, and cultural functions to enhance street vitality and safety. Furthermore, to prevent threshold effects from solely boosting vitality indices, attention must be paid to strengthening regional infrastructure. Attracting investment and developing emerging industries can elevate the city’s economic dynamism and wealth levels, thereby enhancing residents’ perceived prosperity and balancing the factors influencing perceived safety.

Regarding traffic safety, improvements can be achieved through optimized street design. This includes widening roads, establishing dedicated pedestrian and vehicular lanes, and enhancing traffic flow conditions to improve residents’ travel experiences and sense of security. Concurrently, in urban governance and transport planning, big data systems can be leveraged to establish integrated urban management frameworks. These systems consolidate resources across multiple departments—including public security, fire services, transport, and environmental protection—to enhance governance efficiency and interdepartmental coordination. Similarly, for defense security, real-time big data monitoring can be employed. By collecting and analyzing multi-source urban data, a citywide security perception monitoring system can be established to track safety conditions in real time. Concurrently, regular assessments of urban security perception levels should be conducted, alongside establishing a resident feedback mechanism to promptly incorporate and implement suggestions from the primary users of urban safety services.

Furthermore, this paper’s comparative analysis of Nanchang’s historic urban district data confirms that medium-sized historic cities like Nanchang face a dilemma in urban renewal: balancing the preservation of old-city vitality and revitalization of historical resources with the need to improve the urban environment and ensure resident safety. Therefore, urban renewal in such medium-sized, culturally dominant cities should prioritize enhancing residents’ safety perception. This involves improving visual factors like street enclosure and sky visibility, ensuring comprehensive infrastructure services, and elevating social governance’s attention to residents’ psychological perceptions—such as vitality and prosperity awareness. Ultimately, this approach aims to enhance the economic and social development of the city while simultaneously increasing the sense of well-being and security among the indigenous population in the core urban areas.

### Limitations and future research

This study has achieved significant progress in theoretical frameworks and methodological innovation. However, current research still lacks detailed quantitative analysis of respondents across different spatial zones. For instance, in the specific analysis of historic urban districts, only bivariate interaction analysis from SHAP machine learning was introduced, without precise algorithms revealing the operational mechanisms of individual factors. This limits the universality of conclusions and prevents accurate identification of multi-source data mechanisms across distinct functional zones.

Concurrently, the incorporation of perceived data enhances the study’s contribution to the field of perceived safety. However, the absence of objective factors such as crime rates and accident rates within the perceived data composition leaves the specific reasons behind perceived contributions unclear. Future research should continue to explore this aspect.

Moreover, within the computational outcomes of the machine learning model, certain variables beyond perceptual factors exhibit negligible influence on perceived safety. This may stem from their effects being overshadowed by highly influential variables such as vitality perception and wealth perception, or necessitate higher-resolution data to capture localized effects.

Subsequent research will construct a “dual-dimensional objective-subjective” safety evaluation system by incorporating objective data calculations for key variables such as perceptions of urban vitality and wealth. This will integrate multi-source data including crime rates and public sentiment, utilizing geographically weighted regression, high-precision street view features, and hybrid models to optimize spatial heterogeneity analysis and capture localized effects. Concurrently, further investigation is warranted into the role of perceived urban safety among factors constraining development beyond the original core districts in such historically dominant medium-sized cities. Subsequent research may incorporate differential analysis of spatial functional zoning, optimize machine learning models, and deepen theoretical mechanisms and technical frameworks to ultimately achieve high-quality urban development guided by “human-centered safety”.

## Conclusion

This study constructs machine learning models to reveal the complex, non-linear influence mechanisms of multi-source urban data on perceived urban safety. It particularly highlights the pivotal roles of perceived urban vitality and perceived wealth, along with their threshold effects and interactive mechanisms.

Findings indicate that: (1) the contribution to urban safety perception involves a complex, integrated mechanism involving multiple interacting factors, with multi-source data variables across different dimensions exhibiting varying degrees of influence and importance; (2) primary variables within multi-source big data demonstrate pronounced nonlinear relationships and interactive effects, while certain key variables exhibit threshold effects. (3) Perceptions of urban vitality and wealth exert an absolute influence on perceptions of urban safety, exhibiting a fundamentally positive correlation. (4) For medium-sized cities with historical legacies, exemplified by Nanchang, their core historic districts face a dilemma of being both urban centers and areas where governance is impeded. Such regions occupy pivotal dual-role positions within the interplay of urban safety factors and warrant heightened attention regarding their significance in governance and development within these cities. (5) As perceived data relates to human psychological perceptions of the environment, and psychological perceptions arise from complex causes, the influence of perceived data ultimately stems from the mutual interaction between key variables.

Specifically, this study integrates multi-source urban data with interpretable machine learning models (XGBoost and SHAP), transcending the linear assumptions of traditional built environment theories. By incorporating psychological perception factors, it establishes a dynamic assessment framework for “human-centered safety” in urban security. Theoretically, this breakthrough transcends conventional linear analytical frameworks, establishing an interpretable paradigm for multi-source data-driven human-centered urban safety research. Practically, it provides a decision-making foundation for precision planning in medium-sized cities characterized by historical and cultural dominance. This discovery not only enriches theoretical research on urban safety perception, offering scientific grounds for urban planning and public safety management, but also underscores the importance of comprehensively considering economic, social, and spatial factors in urban development. Simultaneously, by innovatively incorporating multidimensional psychological perception data into the study of urban safety perception, this research fills a gap in previous studies that lacked psychological factors. Looking ahead, with the introduction of more high-precision data and the deepening of cross-disciplinary collaboration, the theoretical framework and methodology of this study are expected to provide broader guidance and reference for the sustainable development of global cities and the enhancement of residents’ quality of life, thereby contributing to the construction of urban environments that are more secure and vibrant.

## Data Availability

The datasets generated and analyzed during the current study are available from the corresponding author upon reasonable request. The data has now been uploaded to the public cloud platform.A copy of the dataset has also been temporarily hosted on Baidu Cloud at: https://pan.baidu.com/s/1f6telBYDDYXM_L3_vN4Xbg? pwd=9ivf.
